# Supplementary material for: Content-rich biological network constructed by mining PubMed abstracts
Source: BMC Bioinformatics. 2004 Oct 8;5:147. doi: 10.1186/1471-2105-5-147 (PMC528731; doi:10.1186/1471-2105-5-147)
Supplement: Additional File 2 — The original results of the above study (non-essential files are deleted to keep the file size under the limit set by BMC bioinformatics). [file 1471-2105-5-147-S2.bz2 › chilibotAdditionalFile2/dip50/644ID8642594E11031/html/F_G.html]

 


 **F** and **G** 
  
Found 14371 abstracts in PubMed, retrieved 50.  
 

 What does Google say? 
 PDF only 
| .edu only 

---

**Interactive relationship** (e.g. stimulation, inhibition, etc)

**Neutral relationship**- Viral attachment proteins, hemagglutinin neuraminidase HN, hemagglutinin HA, or glycoprotein  **G** , bind receptors while fusion  **F**  proteins direct membrane fusion.  Ref: 12873767 Biochim Biophys Acta, 2003
- Mice died from GVHD in group  **G**  were significantly more than those in group E and group  **F**  p <.  Ref: 12864951 Zhonghua Xue Ye Xue Za Zhi, 2003
- SpoIIAB, an anti sigma factor involved in regulation of sigma  **F** , is also shown to form a complex with sigma  **G**  in vitro.  Ref: 12867473 J Bacteriol, 2003
- To overcome this inconvenience we aimed to generate intracellular pools of factor  **F**  IX in cells that are implicated in the hemostatic response, e. **g** . endothelial cells and platelets.  Ref: 12871503 J Thromb Haemost, 2003
- factor  **F**  VIII C > 5% but may arise following intense FVIII exposure, e. **g** . continuous infusion CI.  Ref: 12871324 J Thromb Haemost, 2003
- Computing beta on the filtered noise signal beta  **f**  provides a frequency specific metric for the non  **G**  noises that is correlated with the additional frequency specific outer hair cell loss produced by the non  **G**  noise.  Ref: 12880050 J Acoust Soc Am, 2003
- In 151 patients with d dimer >500 micro  **g**  L 1, d dimer, fibrinogen, and d dimer fibrinogen ratio D  **F**  ratio were different in PE positive compared with PE negative patients medians and ranges for d dimer 3793 780 42 195 vs. 992 621 6957 micro  **g**  L 1, fibrinogen 3.8 0.4 6.2 vs. 4.7 2.2 8.4  **g**  L 1, and D  **F**  ratio 1.22 0.15 85.45 103 vs. 0.25 0.09 1.03 x 103.  Ref: 12871404 J Thromb Haemost, 2003
- To achieve these aims, male rats 275 to 300  **g**  were fed 1 of 4 diets for a 4 month period 1 high fat sucrose HFS, 2 low fat complex carbohydrate LFCC, 3 HFS 25 mg kg folic acid HFS  **F** , or 4 LFCC 25 mg kg folic acid LFCC  **F** .  Ref: 12870157 MetabolismMetabolism, 2003
- In mice orally administrered  **F** . fructus 100 microg  **g**  for 1 h, compound 48 80 induced oedema and vascular permeability were significantly reduced rather than those receiving intravenous injection of ketotifen, mast cell stabilizer.  Ref: 12875366 Inflammation, 2003

**Non-interactive relationship** (e.g. studied together, co-existance, homology, etc.)

- We report here the generation of a soluble form of TXAS by deletion of the amino terminal membrane anchoring domain and replacement of the helix  **F**  and  **F**   **G**  loop region with the corresponding region of the structurally characterized microsomal P450 2C5.  Ref: 12859980 Arch Biochem Biophys, 2003
- Compared with traditional heating, central heating significantly increases the risk of exposure to Der  **f**  1 levels > ; 2 μ **g**   **g**  of dust odds ratio, 7.35.  Ref: 12859558 AllergyAllergy, 2003
- The ability of bPIV3 to function as a virus vaccine vector was explored further by introducing the RSV attachment  **G**  and fusion  **F**  genes into the bPIV3 RNA genome.  Ref: 12867647 J Gen Virol, 2003
- Over 2, and 16 wk, on alternate days, mice were treated with an intraperitoneal injection of either 1  **g** .kg 1.day 1 of human intact immunoglobulin or  **F**  ab 2 fragments of human immunoglobulin.  Ref: 12860569 Am J Physiol Heart Circ Physiol, 2003
- However ROC based tests are less powerful when location scale differences exist producing ROC curves that cross the diagonal and are incapable of discriminating between healthy and diseased samples when theta=0.5 but  **F**  not equal  **G** .  Ref: 12872305 Stat Med, 2003
- An increase in the expression of several protease inhibitors with affinity for proteases that promote apoptosis in neutrophils e. **g** ., cystatin  **F** , secretory leukocyte protease inhibitor, as well as the up regulation of a number of antiapoptotic and survival factors was found e. **g** ., adrenomedullin, p38 mitogen activated protein kinase.  Ref: 12873988 Cancer ResCancer Res, 2003
- This recombinant virus expressed the RSV  **G**  and  **F**  proteins sufficiently to evoke a protective immune response in hamsters upon challenge with RSV or human PIV3 and to elicit RSV neutralizing and PIV3 haemagglutinin inhibition serum antibodies.  Ref: 12867647 J Gen Virol, 2003
- A semi parametric approach is based on a completely specified distribution of marker measurements for either the healthy  **F**  or diseased  **G**  subjects, and this is extended to the two marker case.  Ref: 12872305 Stat Med, 2003
- Protein Expression and Peptide Binding Suggest Unique and Interacting Functional Roles for HLA E,  **F** , and  **G**  in Maternal Placental Immune Recognition.  Ref: 12874228 J Immunol, 2003
- Some optimal physicochemical characteristics e. **g** . solvent exposed area, solubility of the molecule may be responsible for this special feature of  **f**  MLP at such a low level of phylogeny.  Ref: 12867161 Cell Biol Int, 2003
- Group A received no treament, group B received TBI only, the rest groups were irradiated 9 Gy by 60 Co and transfused 10 5 CD 34 cells or mixed with 10 7 E, 10 8  **F** , 1.5 x 10 8  **G**  of CD 3 cells respectively.  Ref: 12864951 Zhonghua Xue Ye Xue Za Zhi, 2003
- No other genotypes such as genotype E,  **F** ,  **G** , H were found.  Ref: 12870004 Zhonghua Shi Yan He Lin Chuang Bing Du Xue Za Zhi, 2003
- Zoccali C, Mallamaci  **F** , Tripepi  **G** , Cutrupi S, Parlongo S, Malatino LS, Bonanno  **G** , Rapisarda  **F** , Fatuzzo P, Seminara  **G** , Stancanelli B, Nicocia  **G** , Buemi M Institute of Biomedicine, Clinical Epidemiology and Pathophysiology of Renal Diseases and Hypertension, Reggio Cal.  Ref: 12859694 J Intern MedJ Intern Med, 2003
- Molecular cloning identified six causative genes FANCA, C, D2, E,  **F** , and  **G**  encoding a multiprotein complex whose precise biochemical function remains elusive.  Ref: 12861027 Mol Cell BiolMol Cell Biol, 2003
- In addition, both index patients were homozygous for a new intron  **F**  14T> **G**  mutation, which was found to reduce the acceptor splicing site prediction score.  Ref: 12876630 Thromb Haemost, 2003
- In this study we focused on the structure and expression of the HLA E,  **F** , and  **G**  class I complexes in placental tissue.  Ref: 12874228 J Immunol, 2003
- Given the recognized role of proinflammatory cytokines in septic shock and of extracellular  **F**  and  **G**  actin in the pathophysiology of multiple organ dysfunction, we have investigated the role of Tbeta 4 in sepsis.  Ref: 12860178 Int ImmunopharmacolInt Immunopharmacol, 2003
